# Supplementary material for: Pharmacist-led Medication Counseling for Patients Undergoing Hemodialysis: A Path to Better Adherence
Source: Int J Environ Res Public Health. 2020 Apr 1;17(7):2399. doi: 10.3390/ijerph17072399 (PMC7177809; doi:10.3390/ijerph17072399)
Supplement: Supplementary file 1 [file ijerph-17-02399-s001.pdf]

Kingdom Of Saudi Arabia
National Guard Health Affairs

Supplementary Material

Table S1. Hemodialysis Patient Satisfaction Survey Regarding Medication Counseling. Lolwa Alabdelmuhsin. Pharmacist at KAMC - NGHA

Dear participant I am a pharmacist Lolwa Alabdelmuhsin doing my master research, and conduct this survey to assess your satisfaction regarding pharmacy services about medication counseling. It is a face-to-face survey, which will take about ten to fifteen minutes to complete it. In case for further inquiries or questions, please contact me on this extension number 19887. Thank you.

Patient serial number: ……………………………………………………………………….

First part:

1. **Gender:**
   - ☐ Male.
   - ☐ Female.
2. **Age:**
   - ☐ 18 to 30 years old.
   - ☐ 31 to 50 years old.
   - ☐ 51 to 75 years old.
   - ☐ More than 75 years old.
3. **What language do you mainly speak:**
   - ☐ Arabic.
   - ☐ English.
   - ☐ Both English and Arabic.
   - ☐ Other ________________
4. **What is the highest grade or level of school you have completed?**

- ☐ Primary school or less.
- ☐ Intermediate school.
- ☐ High school.
- ☐ Bachelors.
- ☐ Advanced degree.

5. **Occupational status:**
   - ☐ Employed.
   - ☐ Unemployed.
6. **Material status:**
   - ☐ Single.
   - ☐ Married.
   - ☐ Divorced.
   - ☐ Widowed.
7. **Number of children:**
   - ☐ None.
   - ☐ Five or less.
   - ☐ Ten or less.
   - ☐ More than ten.
8. **Has any kind of co-morbidities:** (You can choose more than one answer)
   - ☐ None.
   - ☐ Hypertension.
   - ☐ Diabetes mellitus.
   - ☐ Other, ____.
9. **For how long have you been on dialysis:**
   - ☐ 3 to 6 months.
   - ☐ More than 6 to 12 months
   - ☐ More than 1 to 5 years.
   - ☐ More than 5 years.

Second part:

1. **I usually claim my medications from:**
   - ☐ Ambulatory Care pharmacy.

- Emergency Pharmacy.

2. **Who supposed to give you the proper information about your prescribed medication:**
    - Physician.
    - Pharmacist.
    - Nurse.
    - Do not know.
3. **The best time for pharmacist counseling during the day of hemodialysis session is:**
    - Before dialysis session.
    - During dialysis session.
    - After dialysis session.
    - Do not know.
4. **Who get your medication usually from the pharmacy:**
    - You.
    - Relatives.
    - House maid or driver.
    - Through the nurse.
    - Other, ____________________.

<u>Third Part:</u> (Rate the following items using a scale of one to ten with ten being Excellent and one being Poor)

Statement	Poor (1)	(2)	(3)	(4)	(5)	(6)	(7)	(8)	(9)	Excellent (10)
The overall pharmacy services for dialysis-dependent patient regarding their prescribed medication	O	O	O	O	O	O	O	O	O	O
The pharmacist counseling regarding your prescribed medication	O	O	O	O	O	O	O	O	O	O

<u>Fourth part:</u>

Statement	Usually	Sometimes	Never	N/A
The pharmacist encourage me to ask any questions regarding my prescribed medication	O	O	O	O
The pharmacist who dispense your prescribed medication listen carefully to you	O	O	O	O
The pharmacist who dispense your prescribed medication explain things in a way you could understand	O	O	O	O
The pharmacist who dispense your prescribed medication give you enough time for counseling	O	O	O	O

Fifth part:

Statement	Agree	Disagree
I prefer to have booklet about my medication	O	O
I think providing hotline for medication counseling will improve my compliance to the medication	O	O
I know that hemodialysis will affect the efficiency of my medications	O	O
I am interested to know more about my medication	O	O
